# Supplementary material for: Semantic and structural image segmentation for prosthetic vision
Source: PLoS One. 2020 Jan 29;15(1):e0227677. doi: 10.1371/journal.pone.0227677 (PMC6988941; doi:10.1371/journal.pone.0227677)
Supplement: S1 Appendix — (ZIP) [file pone.0227677.s001.zip › Revised_SupportingInformation_with_Track_Changes.pdf]

Supplementary material for "Semantic and structural image segmentation for prosthetic vision"

Melani Sanchez-Garcia, Ruben Martinez-Cantin, Jose J. Guerrero
Instituto de Investigación en Ingeniería de Aragón (I3A).
Universidad de Zaragoza, Spain

1 Phosphene generation

In order to simulate the visual perception in prosthetic vision, our phosphenes generated are idealized representations of the percepts feasible in the current implants. Concretely, our phosphene map configuration is similar to the framework described in [1]. First, once the image has been processed according to the chosen processing method, the image is sampled at each location in a given phosphene layout. The most commonly phosphene patterns adopted by SPV in the literature [2] are square or hexagonal array. Due to the prevalence of vertical lines in indoor scenarios [3] we opted to use a square pattern (see Fig ~~??~~1). The simulation of indoor images with square pattern keeps the structure of each line as it really is, while the hexagonal pattern tends to distort it due to the deviation of the phosphenes when forming hexagons (Fig ~~??~~ 1 (right)).

Fig 1. Vertical lines in indoor environments. (a) ~~Example~~ Prevalence of ~~indoor scene, (b) prevalence of~~ vertical lines, (~~c~~b) square pattern and (~~d~~c) hexagonal pattern. As a result of the abundance of vertical lines in indoor environments, we opted to use the square grid.

Similarly to many SPV studies [2], phosphenes are approximated as grayscale circular dots with a Gaussian luminance profile. The luminance profile of each phosphene has maximum intensity at the center and gradually decays to the periphery, following an unnormalized Gaussian function $G(x, y)$ defined in Eq (1).

$$G(x,y) \propto \exp\left\{\frac{(x-\mu_x)^2+(y-\mu_y)^2}{2\sigma^2 lum}\right\} \tag{1}$$

The intensity of a phosphene is a function $f : \mathbb{R}^n \rightarrow \mathbb{R}$ of the intensity of the pixels covering the same location in the processed image. Usually, f is the mean function, but we found that the median function preserved the line structures of SIE-OMS and Edges. In addition, each sampled pixel intensity (i) is quantified to each individual phosphene's dynamic range as:

$$lum(x,y) = \frac{i}{max(l)} \tag{2}$$

In Eq (2), l is the number of gray levels intensity of the phosphene. The size and brightness are directly proportional to the quantified sampled pixel intensities. Then, the phosphene profile $P(x,y)$ is applied to every phosphene resulting in the final image:

$$P(x,y) = lum \cdot G(x,y) \tag{3}$$

where $P(x,y)$ represents the pixel value at the coordinates (x,y) of the stimulus image (Eq (3)).

Furthermore, several important works have demonstrated that exists a degree of incomplete phosphene visual field maps (dropout) in implanted patients. This is because of the high threshold required values to elicit phosphenes that placed in areas with a high proportion of dead nerve cells [4]. This results in a lower resolution than the number of electrode elements. We included a 10% dropout of phosphenes. Those phosphenes were initially selected randomly but kept the same for all the images. Note that our prosthetic vision simulation does not integrate all aspects of visual appearance reported by implanted subjects (e.g. distortion effect [5, 6]).

References

[1] McKone E, Robbins RA, He X, Barnes N. Caricaturing faces to improve identity recognition in low vision simulations: How effective is current-generation automatic assignment of landmark points? PloS one. 2018;13(10):e0204361.

[2] Chen SC, Suaning GJ, Morley JW, Lovell NH. Simulating prosthetic vision: I. Visual models of phosphenes. Vision Research. 2009;49(12):1493–1506.

[3] Chu J, GuoLu A, Wang L, Pan C, Xiang S. Indoor frame recovering via line segments refinement and voting. In: IEEE International Conference on Acoustics, Speech and Signal Processing; 2013. p. 1996–2000.

[4] Humayun MS, Weiland JD, Fujii GY, Greenberg R, Williamson R, Little J, et al. Visual perception in a blind subject with a chronic microelectronic retinal prosthesis. Vision Research. 2003;43(24):2573–2581.

[5] Guo H, Wang Y, Yang Y, Tong S, Zhu Y, Qiu Y. Object recognition under distorted prosthetic vision. Artificial Organs. 2010;34(10):846–856.

[6] Cao X, Li H, Lu Z, Chai X, Wang J. Eye-hand coordination using two irregular phosphene maps in simulated prosthetic vision for retinal prostheses. In: IEEE 10th International Congress on Image and Signal Processing, BioMedical Engineering and Informatics; 2017. p. 1–5.
