# Supplementary figures and images for: Semantic and structural image segmentation for prosthetic vision
Source: PLoS One. 2020 Jan 29;15(1):e0227677. doi: 10.1371/journal.pone.0227677 (PMC6988941; doi:10.1371/journal.pone.0227677)

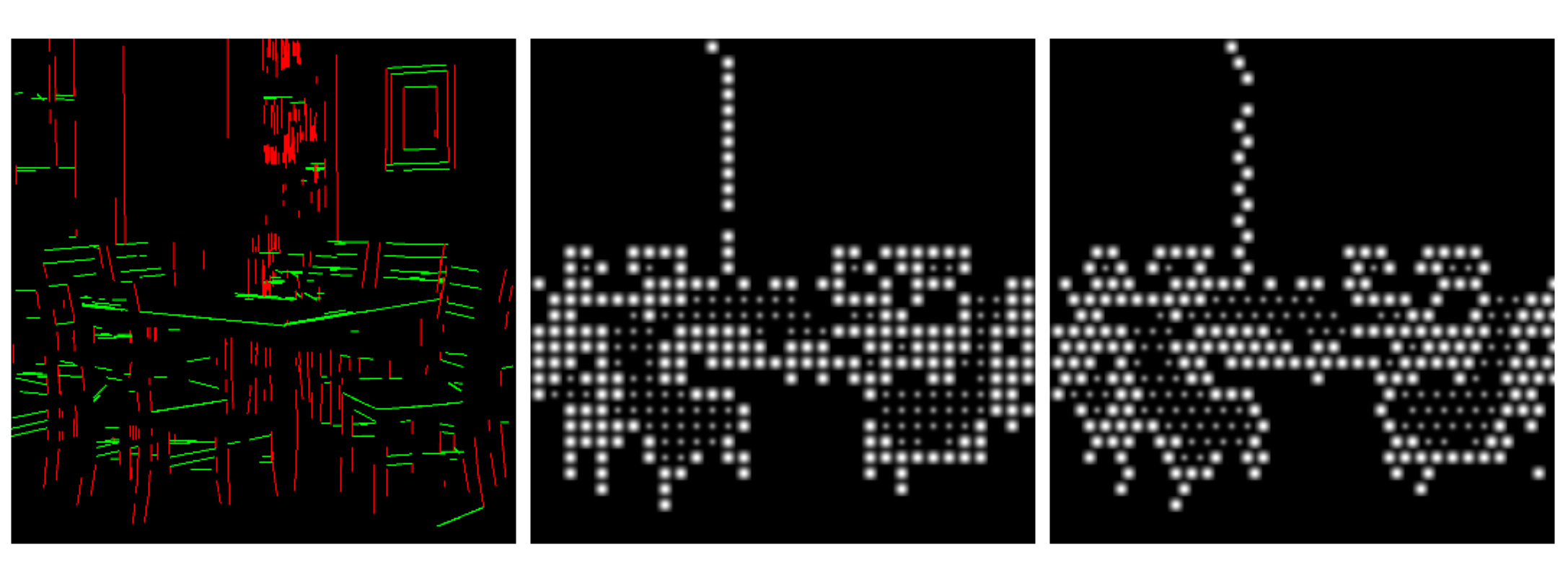

Supplement: S1 Appendix — (ZIP) [file pone.0227677.s001.zip › Fig1.tiff]
